# Step-in dosing of bosutinib in pts with chronic phase chronic myeloid leukemia (CML) after second-generation tyrosine kinase inhibitor (TKI) therapy: results of the Bosutinib Dose Optimization (BODO) Study

**DOI:** 10.1007/s00277-023-05394-0

**Published:** 2023-08-18

**Authors:** Susanne Isfort, Kirsi Manz, Lino L. Teichmann, Martina Crysandt, Andreas Burchert, Andreas Hochhaus, Susanne Saussele, Alexander Kiani, Joachim R. Göthert, Thomas Illmer, Philippe Schafhausen, Haifa Kathrin Al-Ali, Frank Stegelmann, Mathias Hänel, Tim Pfeiffer, Aristoteles Giagounidis, Georg-Nikolaus Franke, Steffen Koschmieder, Alice Fabarius, Thomas Ernst, Mareille Warnken-Uhlich, Uta Wolber, Denise Kohn, Markus Pfirrmann, Dominik Wolf, Tim H. Brümmendorf, for the German CML study group

**Affiliations:** 1 Department of Hematology, Oncology, Hemostaseology and Stem Cell Transplantation, Medical Faculty, RWTH Aachen University, Aachen, Germany; 2 Center for Integrated Oncology Aachen Bonn Cologne Düsseldorf (CIO ABCD), Aachen Bonn Cologne Düsseldorf, Germany; 3 Institute for Medical Information Processing, Biometry and Epidemiology (IBE), Faculty of Medicine, LMU Munich, Munich, Germany; 4 Institute for Community Medicine, University Medicine Greifswald, Greifswald, Germany; 5 Department of Medicine III, University Hospital Bonn, Bonn, Germany; 6 Dep. of Internal Medicine, Hematology, Oncology and Immunology, Philips Univ. Marburg, Marburg, Germany; 7 Hematology/Oncology, Universitätsklinikum Jena, Jena, Germany; 8 Department of Hematology and Oncology, University Hospital Mannheim, Heidelberg, Germany; 9 Department of Oncology and Hematology, Klinikum Bayreuth, Bayreuth, Germany; 10 Comprehensive Cancer Center Erlangen-EMN (CCC ER-EMN), Erlangen, Germany; 11 Department of Hematology and Stem Cell Transplantation, West German Cancer Center, University Hospital Essen, University of Duisburg-Essen, Essen, Germany; 12 Hematology-Oncology Practice, Dresden, Germany; 13 Department of Oncology, Hematology and Bone Marrow Transplantation With Section of Pneumology, University Medical Center Hamburg-Eppendorf, Hamburg, Germany; 14 University Hospital Halle, Halle (Saale), Germany; 15 Department of Internal Medicine III, University Hospital of Ulm, Ulm, Germany; 16 Department of Internal Medicine III, Küchwald Hospital Chemnitz, Chemnitz, Germany; 17 Department of Hematology and Oncology, Klinikum Augsburg, Augsburg, Germany; 18 Clinic for Oncology, Hematology, and Palliative Medicine, Marien Hospital Düsseldorf, Düsseldorf, Germany; 19 Medical Clinic I, University Hospital of Leipzig, Leipzig, Germany; 20 Clinical Study Core Unit Bonn, Institute of Clinical Chemistry and Clinical Pharmacology, University Bonn, Bonn, Germany; 21 Internal Medicine V, Department for Hematology and Oncology, Comprehensive Cancer Center Innsbruck (CCCI), Tyrolean Cancer Research Institute (TKFI), Medical University of Innsbruck (MUI), Innsbruck, Austria

**Keywords:** CML, Bosutinib, Gastrointestinal toxicity, Dose escalation, Efficacy

## Abstract

The approved dose of bosutinib in chronic phase CML is 400 mg QD in first-line and 500 mg QD in later-line treatment. However, given that gastrointestinal (GI) toxicity typically occurs early after treatment initiation, physicians often tend to start therapy with lower doses although this has never been tested systematically in prospective trials in the Western world. The Bosutinib Dose Optimization (BODO) Study, a multicenter phase II study, investigated the tolerability and efficacy of a step-in dosing concept of bosutinib (starting at 300 mg QD) in chronic phase CML patients in 2^nd^ or 3^rd^ line who were intolerant and/or refractory to previous TKI treatment. Of 57 patients included until premature closure of the study due to slow recruitment, 34 (60%) reached the targeted dose level of 500 mg QD following the 2-weekly step-in dosing regimen. While the dosing-in concept failed to reduce GI toxicity (grade II–IV, primary study endpoint) to < 40% (overall rate of 60%; 95% CI: 45–74%), bosutinib treatment (mean dosage: 403 mg/day) showed remarkable efficacy with a cumulative major molecular remission (MMR) rate of 79% (95% CI: 66 to 88%) at month 24. Of thirty patients refractory to previous therapy and not in MMR at baseline, 19 (64%) achieved an MMR during treatment. GI toxicity did not significantly impact on patient-reported outcomes (PRO) and led to treatment discontinuation in only one patient. Overall, the results of our trial support the efficacy and safety of bosutinib after failure of second-generation TKI pre-treatment. Trial registration: NCT02577926.

**Supplementary Information:**

The online version contains supplementary material available at 10.1007/s00277-023-05394-0.

## Introduction

Bosutinib is a second-generation TKI approved in Europe in 2013 for CML-chronic phase (CP), accelerated phase (AP), and blast crisis (BC). The licensed starting dose of bosutinib has recently been defined to be 400 g QD for first [1, 2] and 500 mg in later lines of CML treatment in both CP and advanced disease stages [3–5]. Approval of bosutinib for CML was based on the results of a phase I/II trial in second- and later-line therapy and of the BFORE trial in first-line therapy [3, 6–9]. In the recently published post-approval BYOND trial in pts in second, third, and fourth line, bosutinib was also able to induce a cumulative MMR rate by 1 year of 70.5% in the overall Philadelphia chromosome (Ph) + CP-CML cohort (TKI-resistant: 60.5%; TKI-intolerant: 80.8%); In pts lacking baseline MMR, the cumulative 1-year MMR rate was 58.2% (TKI-resistant: 43.8%; TKI-intolerant: 80.6%) [10]. When focusing only on second-line bosutinib pts, the 1- and 2-year MMR rates were 80.4% and 82.6%, respectively. Notably, 35 out of 46 second-line pts were “only” pre-treated with imatinib [10]. Thus, only very few data on the effectiveness of second-line bosutinib therapy after failure/intolerance of previously given second-generation TKI (i.e., nilotinib or dasatinib) are available.

The most frequent side effects of bosutinib treatment are gastrointestinal toxicities. In the BYOND trial, 87.7% suffered from all grade diarrhea (comprising 16% grade 3/4), 39.9% from nausea, and 32.5% from treatment-emergent vomiting mostly in the initial days of treatment [10]. Even in the BFORE trial where bosutinib was administered at a lower dose of 400 mg QD, diarrhea occurred in 70.1% of the pts (comprising 7.8% grade 3/4 diarrhea) [1, 2].

Generally speaking, gastrointestinal side effects are very common across all bosutinib trials but typically remain manageable [11] with either transient concomitant medication, dose interruption, and/or dose reductions [7] thereby enabling long-term bosutinib treatment with only very few permanent treatment discontinuations [1, 10].

Preservation of efficacy following secondary dose reductions of bosutinib (after initiation of treatment with the approved standard dose) has mostly been retrospectively addressed within the study populations assessed showing similar efficacy even in lower dosages [12, 13].

The purpose of the current phase 2 study was to evaluate whether a new therapeutic scheduling approach, termed “bosutinib step-in dosing regimen,” might be able to decrease early-occurring GI toxicity while maintaining optimal efficacy according to ELN recommendations [14] in pts with CML after failure or intolerance to previous TKI therapy.

## Methods

### Study design and pts

The BODO trial (NCT02577926; CML-7 Study) is a multicenter, open label, single-arm, non-randomized phase II trial testing the tolerability and efficacy of 2nd and 3rd line bosutinib step-in dosing in CP CML pts intolerant and/or refractory to previous TKI therapy. Eligible pts were adults with a cytogenetic or qualitative polymerase chain reaction (PCR)–based diagnosis of Ph + and/or BCR::ABL1 + CP-CML, prior treatment with one or maximum two lines of TKI treatment for CML, and adequate hepatic/renal function (for details, please find attached the study protocol in the supplement of this article). An initial pre-treatment with imatinib for up to 6 weeks did not count as an autonomous line of therapy. Pts were required to have Eastern Cooperative Oncology Group performance status (ECOG PS) 0 or 1; pts with leptomeningeal leukemia or a known BCR::ABL1 T315I or V299L mutation were excluded. Intolerance to prior therapy was defined as discontinuation of Imatinib OR Nilotinib OR Dasatinib due to grade 3– or 4–related adverse event (AE), despite optimal supportive care, or because of a persistent grade 2–related AE, despite optimal supportive care, which persisted ≥ 1 month or recurred > 2 times with TKI dose reduction or which was medically significant (independent of grade) and according to investigator’s opinion lead to change of TKI. Resistance to prior therapy was defined as not achieving optimal response to Imatinib OR Nilotinib OR Dasatinib according to ELN2013-defined recommendations [15].

Bosutinib was commenced with 300 mg QD and was (in the absence of > G 1 toxicities) dose-increased by increments of 100 mg daily dosing every 14 days up to a maximum target dose of 500 mg QD. Consecutive dose reductions to 400, 300, or 200 mg QD due to toxicity/tolerability were permitted. Special recommendations regarding management of bosutinib-related diarrhea were given to physicians and patients and early use of prophylactic medication such as loperamide after onset of diarrhea was encouraged. Planned observation was 2 years from the time of first dose, unless disease progression, unacceptable toxicity, withdrawal of consent, death, or study discontinuation.

Initially recruitment of 127 subjects who would have received at least 14 daily doses of study medication was planned to ensure sufficient power for a reasonable assessment of grade 2–4 GI toxicity as the primary endpoint. Sample size calculation and determination of the exact CI were based on Chow and colleagues [16]. However, due to slow recruitment, the trial had to be stopped prematurely after inclusion of 57 pts.

### Endpoints and analyses

#### Primary endpoint

The primary endpoint was the incidence rate of grade 2 to 4 GI toxicity independent of relatedness to the study drug within 6 months after registration. The null hypothesis of the trial was defined as bosutinib leading to GI toxicity grades 2 to 4 in ≥ 40% of the pts. All pts were asked and examined for grade 2 to 4 GI toxicity at their individual 6-month visits. Pts were evaluable, if they received at least 14 daily doses of study medication and either had a grade 2 to 4 GI toxicity at any time within the first 6 months or were observed for the complete 6 months without experiencing any grade 2 to 4 GI toxicity. To be included in the analysis, pts without previously reported grade 2 to 4 GI toxicity should have had an examination within the interval around 6 months, i.e., between > 4.5 and 9 months, but preferably between 6 and 9 months to cover at least 6 months observation time. This primary endpoint was confirmatively tested. The 95% confidence interval (CI) around the estimated rate of the primary endpoint was calculated in accordance with Clopper and Pearson [17].

#### Further safety analysis

AEs and SAEs that occurred during the study treatment until 28 days after the last administration of the last dose of study medication were recorded (TEAEs). The time-to-AE analysis was performed using the Aalen-Johansen estimator [18] which allows calculation of cumulative incidence probabilities over time under consideration of competing risks [19]. Competing risks were all events observed before a possible observation of grade 2 to 4 GI toxicity and preventing a later observation of grade 2 to 4 GI toxicity: death, progression of disease, or other serious adverse events leading to treatment discontinuation.

#### Patient-reported outcome measures (PROM)

The EORTC QLQ-C30 and its CML module QLQ-CML-24 questionnaire were scored according to the respective user’s guides. Summary statistics of the quality of life questionnaire were calculated at baseline, month 3, and month 6.

For further details on methods, please see supplemental file F1. Data are from the locked trial database with a cut-off date of January 28, 2021.

## Results

### Pts and treatment

**Fig. 1:**
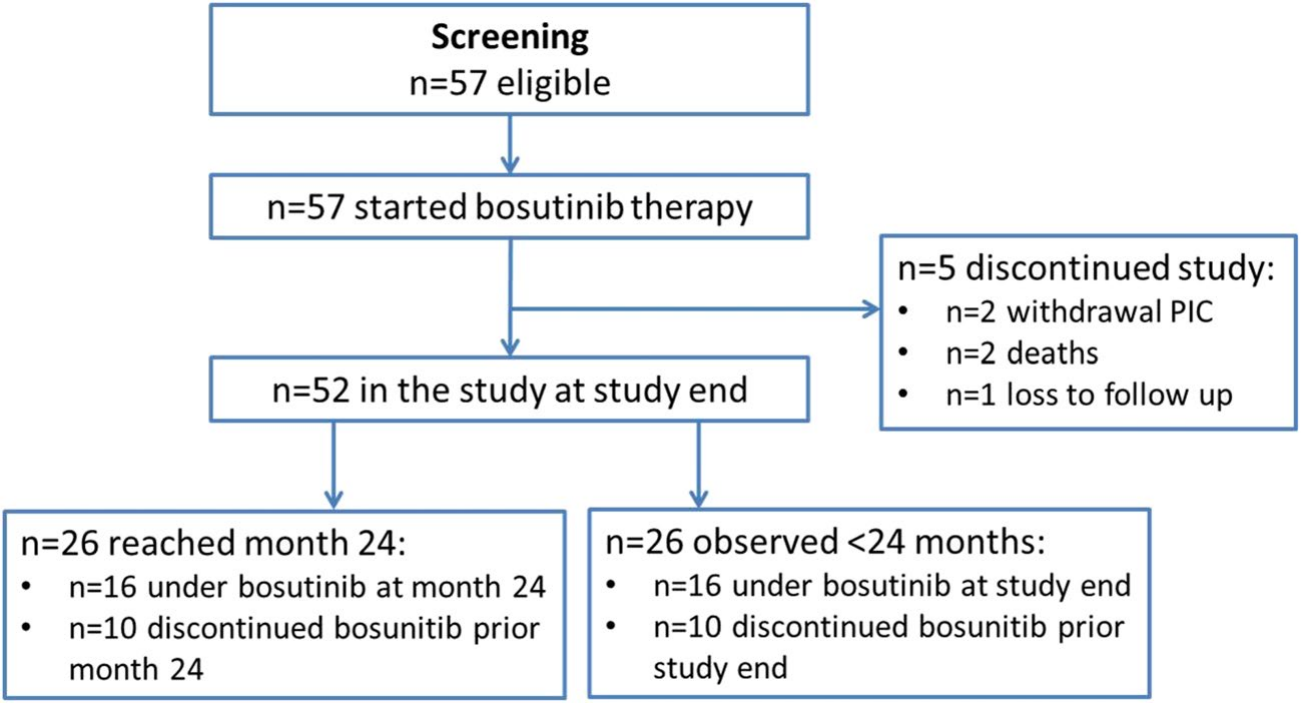
CONSORT diagram for the BODO trial (PIC, patient’s informed consent)

A total of 57 pts with Ph + CML in first CP were enrolled between April 2016 and December 2019 across 20 study centers of the German CML Study Group (for pts’ disposition, see Fig. 1). The pts were followed for a median time of 22 months (range: 1–46). Fifty-six percent (*n* = 32) of pts were male, and the median age was 51 years (range: 19–77; for detailed pts’ characteristics, see Table 1). The study was prematurely stopped after the last enrolled patient had concluded 6 months of study participation; 46% (*n* = 26) of pts had ≥ 2-year follow-up. Twenty-five pts discontinued bosutinib prematurely (before reaching 24 months of treatment within the study), seventeen (68%) due to an AE and 5 (20%) due to insufficient clinical response. Three pts (out of 25; 12%) discontinued bosutinib due to other reasons (one case of pregnancy, one case of stem cell transplantation, and one case of withdrawal of patient´s informed consent). Median duration of bosutinib treatment overall was 15 months (range: 0–44): 18 months (0–44) in the second-line and 9 months (0.5–36) third-line cohorts, respectively. Median duration of bosutinib treatment was 16 months (range: 0–44) in TKI-resistant and 14 months (range: 0–42) in TKI-intolerant pts, respectively. PROMs were assessed in 51, 44, and 35 of the 57 pts at baseline, month 3, and month 6, respectively. All available measurements were used to calculate PROMs.

**Table 1:** Baseline characteristics of all (*n* = 57) patients (median observation time: 21.7 months)

	*N*	Median	Min	Max
Age (years)	57	51.0	19.0	77.0
Blasts (%)	42	0.0	0.0	1.0
Spleen size (cm below costal margin)	53	0.0	0.0	0.0
Spleen size (cm)	41	10.0	8.0	16.0
Leukocytes (10^9/l)	57	5.9	2.5	12.7
Platelets (10^9/l)	57	207	44	641
Hemoglobin (mmol/l)	57	8.7	5.8	10.4
Pre-treatment duration (with any TKI)	56	20	1	129
Pre-treatment duration				
Intolerant pts	22	17	1	126
Refractory pts	34	20	2	129
Pre-existing medical conditions	57	3	0	18
	*N*	%		
Gender (male)	32	56		
ECOG performance status				
0	45	79		
1	12	21		
TKI pre-treatment	Frequency			Percentage
Pre-treatment before bosutinib as second-line therapy				
Nilotinib	23			40%
Dasatinib	22			39%
Imatinib	4			7%
Pre-treatment before bosutinib as third-line therapy				
Imatinib + Dasatinib	2			4%
Imatinib + Nilotinib	2			4%
Dasatinib + Nilotinib	2			4%
Imatinib + Nilotinib + Dasatinib	2			4%
Response to previous therapy				
Intolerant	23			40%
Refractory	34			60%

### Bosutinib dosing

**Fig. 2:**
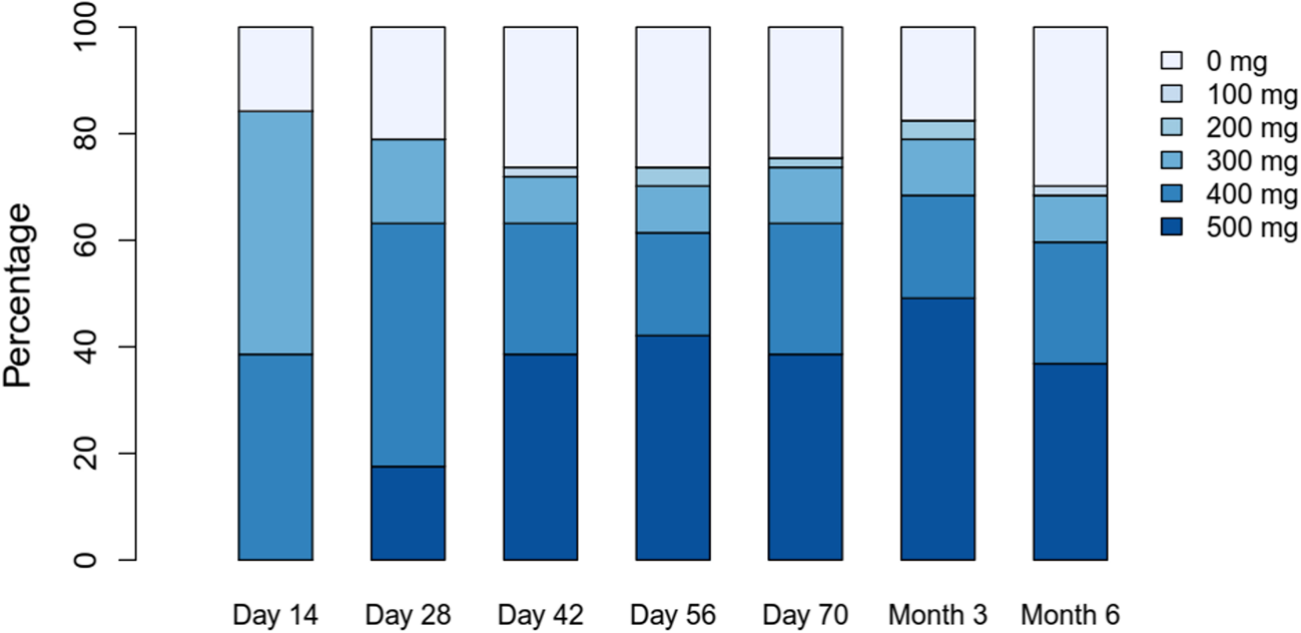
Distribution of all 57 pts to different dosing levels of bosutinib from baseline until month 6; mg, milligram

Core element of the study was the evaluation of the step-in dosing concept. The maximum duration of the dose optimization period was 3 months. If a patient was not able to enter the highest dose level of 500 mg at month 3, the current dose level was to be administered for the rest of the study (unless new side effects or events occurred that required dose reduction). All 57 pts started at day 1 with 300 mg bosutinib (QD). Thirty-four (60%) pts successfully completed the 2-weekly step-in dosing scheme and reached the targeted dose level of 500 mg QD (for distribution of pts among the different dosing levels at all visits until month 3, see Fig. 2). However, of these 34 pts, 2 (6%) pts discontinued bosutinib within the first 3 months of treatment and further 4 (12%) pts had to step back to 400 mg bosutinib at month 3 based on side effects. Overall, twenty-three pts (40%) did not reach the maximum dose level of 500 mg QD at the end of the dose escalation phase of the study. Of these 23 pts, 7 (30%) were treated with 400 mg bosutinib, 6 (26%) with 300 mg, 2 (9%) with 200 mg, and 8 (35%) had discontinued bosutinib within the first 3 months. Two pts were later dose-escalated to 500 mg bosutinib (one because of lack of efficacy and the other for unknown reasons) and stayed at that dose level. Factors associated with successful dosing in (*n* = 34 pts) were evaluated in a multivariate analysis revealing body weight to be a factor of special interest. Per increase of one unit in BMI, the odds for successful step-in dosing increased by 10% to 1.1 (1.0–1.3). Per 10 kg increase in weight, the odds for successful step-in dosing increased by 70% to 1.7 (1.2–2.4).

### Safety

**Fig. 3:**
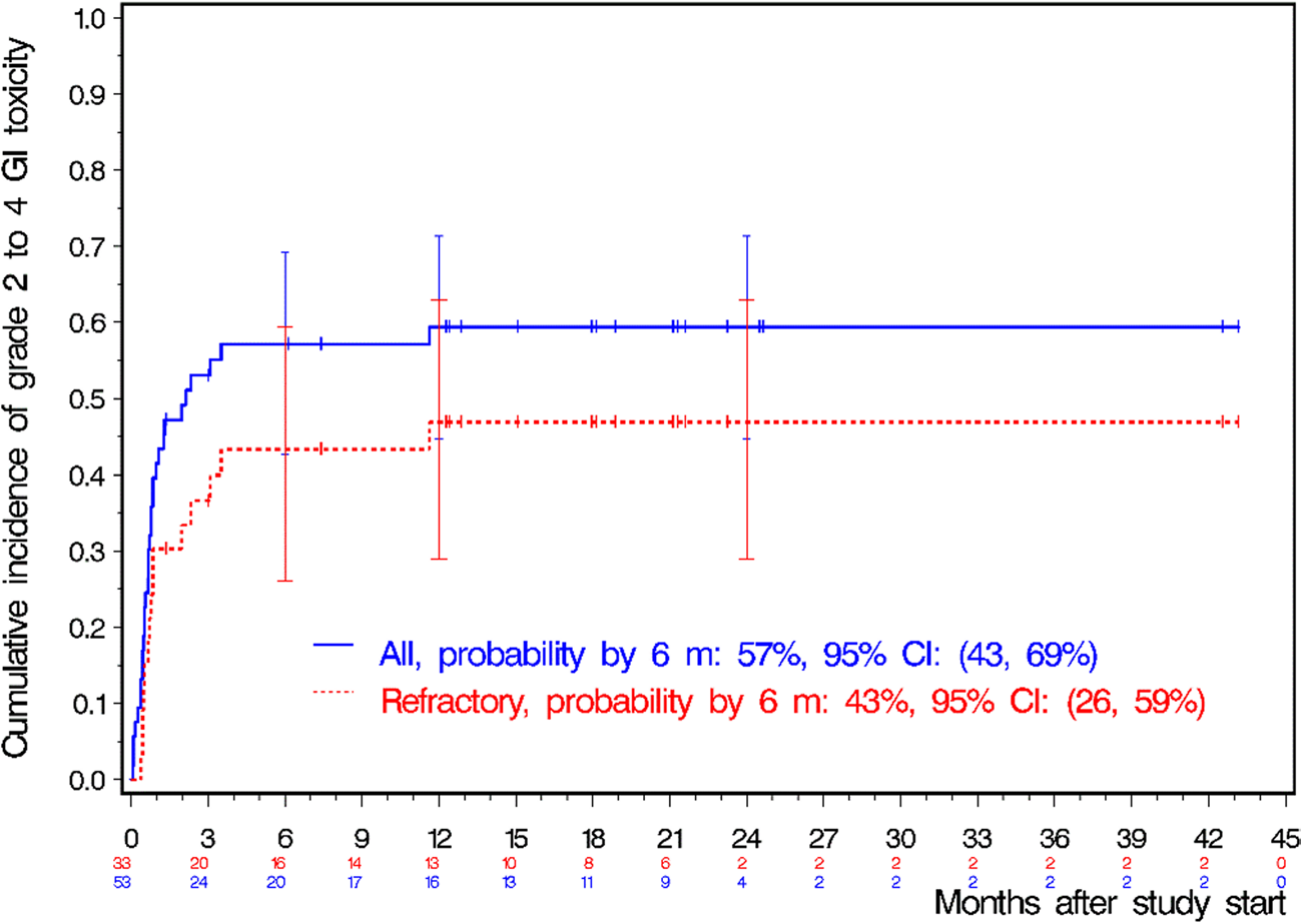
Cumulative incidence of grade 2 to 4 gastrointestinal (GI) toxicity in the primary analysis data set (*n* = 53, All) and for pts refractory to former treatment (Refractory); m, months; CI, confidence interval

The primary analysis data set included 53 pts who received at least 14 daily doses of bosutinib. Per protocol, four pts receiving bosutinib only for 11, 12, 12 and 10 days, respectively, were excluded from further analysis. Three additional pts had to be excluded due to an insufficient observation time (i.e., < 6 months). As a consequence, 50 out of the total of 57 enrolled pts were eligible for primary endpoint analysis. Twenty pts (40%) did not develop any clinically relevant GI toxicity > grade 1 during the first 6 months including 6 pts (11%), who did not develop any grade of GI toxicities. The overall rate of grade 2 to 4 GI toxicity within the first 6 months of treatment was 60% (95% CI: 45–74%). Thus, the null hypothesis of the trial (GI toxicity was assumed to be reduced to < 40%) could not be discarded. Remarkably, GI toxicity led to treatment discontinuation in only one patient keeping in mind that only 60% could successfully increase the dose to 500 mg. Rates of grade 2 to 4 GI toxicity within the first 12 and 24 months of treatment in the primary analysis data set were 65% and 72%, respectively, showing that most of the higher grades GI toxicity happened during the first six months (see Fig. 3). Two pts died upon study inclusion, one due to CML progression in a later line of therapy (no MMR with bosutinib, death 6 months after allogenic stem cell transplant) and one patient due to bleeding of a cerebral cavernoma which was judged to be unrelated to the study drug.

Adverse events led to dose reduction in 53% (*n* = 30) and to treatment interruption in 54% (*n* = 31) of pts. Seventeen out of 25 pts who discontinued their treatment early did this because of an AE (30% of all pts). Eleven patients discontinued treatment during the first 3 months corresponding to the dose escalation phase. Ten out of 11 patients discontinued because of adverse events that were affecting dose escalation. The rate of dose reductions due to AEs was 47% (*n* = 16) in TKI-resistant and 61% (*n* = 14) in TKI-intolerant pts; the rates of temporary interruptions due to AEs were 47% (*n* = 16) and 65% (*n* = 15), respectively.

The most common AEs leading to discontinuation were elevation of liver enzyme serum levels (i.e., ALT increased *n* = 5, AST increased *n* = 4) or increased gamma-glutamyltransferase (*n* = 3). The most frequently reported AEs in the overall patient population were diarrhea (*n* = 42 pts, 74%), nausea (*n* = 31, 54%), and ALT increase (*n* = 24, 42%). All frequently AEs (> 30%) and AEs of grade 3/4 AEs occurring in > 5% of pts can be found in Table 2.

**Table 2:** The most frequently reported adverse events including corresponding G3/4 AEs and details on GI toxicity

AE*	All grades		Grades 3/4	
	No. of patients	%	No. of patients	%
Diarrhea	42	74	8	14
Nausea	31	53	1	2
Alanine aminotransferase increased	24	42	10	18
Fatigue	21	37	3	5
Headache	19	33	1	2
Abdominal pain	17	30	2	4
Aspartate aminotransferase increased	17	30	5	9
Vomiting	15	26	2	4
Gamma-glutamyltransferase increased	12	21	2	4
Rash	9	16	1	2
Dizziness	8	14	0	0
Electrocardiogram QT prolonged	8	14	1	2
Abdominal pain upper	7	12	1	2
Arthralgia	7	12	0	0
Blood creatinine increased	6	11	0	0
Platelet count decreased	6	11	0	0
Pyrexia	6	11	0	0
Thrombocytopenia	6	11	2	4
Median duration of GI toxicity in days	15	Range (1–1281)		
Median time to first AE of diarrhea in days	23	Range (3–53)		
Median duration of any grade of diarrhea in days	14	Range (1–960)		

Legend: *According to the MedDRA Preferred Term; in the overall patient population (*n* = 57), all pts had ≥ 1 any grade adverse event and 72% (*n* = 41) of pts had ≥ 1 grade 3/4 AE

### Efficacy

**Fig. 4:**
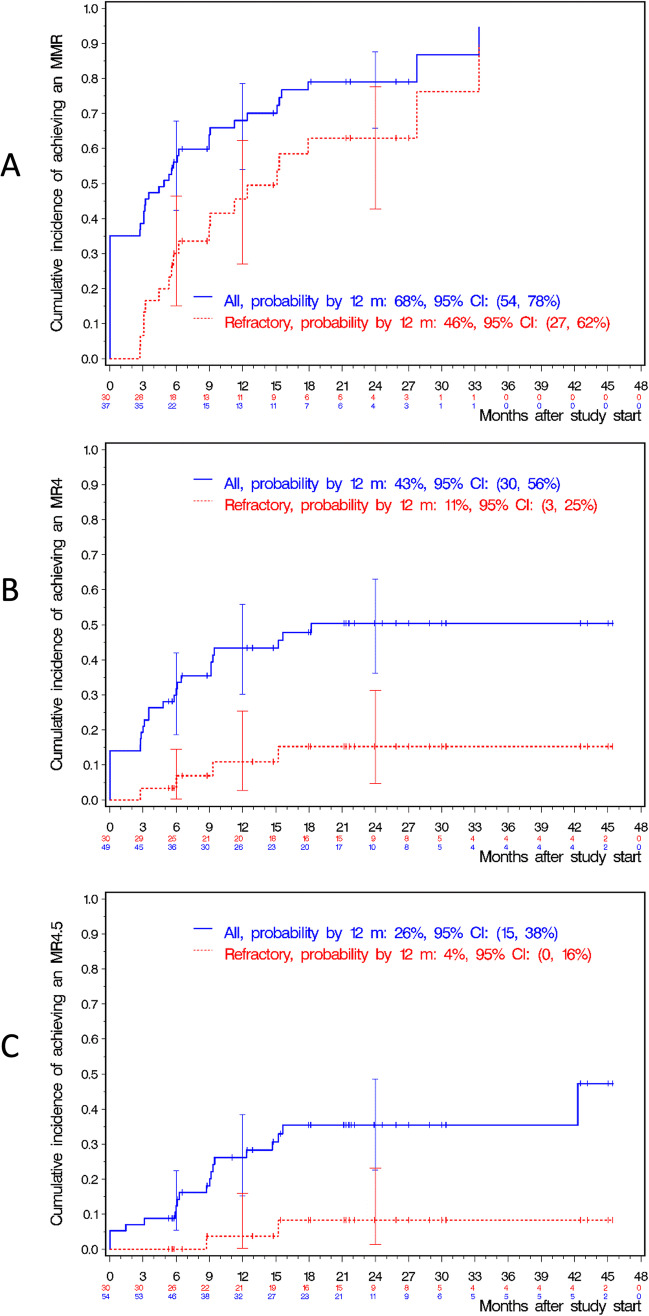
Probabilities of MMR, MR^4^, and MR^4.5^ for the whole study cohort (All) and for patient refractory to former treatment (Refractory). **A** MMR rate; **B** MR^4^ rate; **C** MR^4.5^ rate; MMR, major molecular remission; MR^4^, deep molecular remission *BCR::ABL1* transcripts ≤ 0.01%; MR4.5, deep molecular remission *BCR::ABL1* transcripts ≤ 0;0032%; m, months; CI, confidence interval

Forty-six pts were evaluable for efficacy parameters. The cumulative confirmed MMR rate (95% CI) by 1 year was 68% (54–78%, see Fig. 4), the MR4 and MR4.5 rates were 43% (30–56%) and 26% (15–38%), respectively.

Six out of 7 intolerant pts without MMR at baseline reached MMR or a better molecular response level with bosutinib. Thirty pts refractory to previous therapy (19 being resistant; 11 being resistant and intolerant) were lacking baseline MMR, of which 19 pts achieved MMR or better (2 pts with MR4.5, 2 with MR4 and 15 with MMR). In the 30 refractory pts without MMR at baseline, the cumulative MMR rate by 1 year was 46% (27–62%) and MR4 and MR4.5 rates were 11% (3–25%) and 4% (0–16%), respectively (Fig. 4). Molecular responses and probabilities of molecular response to bosutinib are depicted in Table 3. Median dose levels of bosutinib were not significantly different between responders and non-responders (see Table 4). Forty pts received bosutinib up to 6 months and also had a 6-month molecular response evaluation. No disease progressions were reported during the study duration and follow-up.

**Table 3:** Molecular response (MR) rates and probabilities of molecular response at 3, 6, 12, 18, and 24 months

Time	MMR rate (95% confidence interval)	MR4 rate (95% confidence interval)	MR4.5 rate (95% confidence interval)
3 months	23/50 = 46% (32, 61%)	11/50 = 22% (12, 36%)	3/50 = 6% (1, 17%)
6 months	31/54 = 57% (43, 71%)	17/54 = 32% (20, 46%)	7/54 = 13% (5, 25%)
12 months	31/46 = 67% (52, 81%)	12/44 = 27% (15, 43%)	8/44 = 18% (8, 33%)
18 months	25/41 = 61% (45, 76%)	13/40 = 33% (19, 49%)	12/40 = 30% (17, 47%)
24 months	16/25 = 64% (43, 82%)	10/25 = 40% (21, 61%)	8/25 = 32% (15, 54%)
Time	Probability of MMR (95% confidence interval)	Probability of MR4 (95% confidence interval)	Probability of MR4.5 (95% confidence interval)
3 months	39% (26–51%)	21% (12–32%)	7% (2–16%)
6 months	56% (42–68%)	30% (19–42%)	12% (6–22%)
12 months	68% (54–78%)	43% (30–56%)	26% (15–38%)
18 months	79% (69–88%)	48% (34–60%)	35% (23–49%)
24 months	79% (66–88%)	50% (36–63%)	35 (23 – 49%)

Legend: *MMR*, major molecular response corresponding to MR3

At baseline, 20 pts (35%) had entered the study in MMR, 8 pts (14%) were already in MR4, and 3 pts (5%) were already in MR4.5. Median time to MMR was 4.4 months and median time to MR4 was 18 months

**Table 4:** Median bosutinib dose according to level of molecular response

Group	Response	N	Min	Median	Max	*p*
MMR	Yes	23	208	460	479	0.4600
	No	17	192	398	478	
MR4	Yes	11	243	455	479	0.7504
	No	29	192	403	478	
MR4.5	Yes	4	300	470	479	0.2411
	No	36	192	402	478	

Legend: Forty patients received bosutinib up to 6 months and also had a 6-month molecular response evaluation (Min, Median Max: minimum, median, and maximum bosutinib dosage within the first 6 months; *MMR*, major molecular remission; MR4 and MR4.5 deep molecular remission)

### PROM during treatment and relation to AEs

In order to investigate the impact of gastrointestinal toxicity on PROMs, PROM results were analyzed according to occurrence of GI toxicity: regarding diarrhea, we divided the pts into two groups: 42 pts who had experienced diarrhea within the first 6 months of treatment and 15 pts without diarrhea. Only the insomnia symptom scale differed significantly between the groups after 6 months (23 vs. 52, *p* = 0.0248) with pts without diarrhea experiencing higher symptom scores. Nausea and vomiting did not lead to a statistically significant difference in symptom burden. As most of these symptoms set on early during treatment period, we speculated that the influence of GI side effects might not be detectable any more at month 6, that is why we performed the same analysis for the 3-month visit. However again, at this visit, neither pts with diarrhea nor nausea or vomiting showed significantly different results in comparison to pts without those side effects.

## Discussion

Overall and despite its premature termination, the results of the BODO trial using step-in dosing of bosutinib confirms the efficacy findings from other later-line bosutinib trials such as the phase I/II trial or the BYOND trial [3, 13, 20, 21]. Seventy-nine percent of the pts included achieved MMR during the study, almost half of them (48%) MR^4^ and every third patient (33%) reached MR^4.5^. In the BYOND study, second-line bosutinib yielded major cytogenetic remission (MCyR), complete cytogenetic remission (CCyR), and MMR rates (both by 24 months) of 80%, 81.3%, and 76%, respectively, in pts without the respective baseline response [10]. In our study, out of the 37 pts without baseline MMR, 25 (68%) achieved MMR or better molecular responses. We did not document baseline cytogenetic remission status in our study. Considering that 68% of pts achieved a molecular response after failure to previous treatment and that 45 of 57 pts in the BODO trial were in a second-line setting after failure to another second-generation TKI in 1st-line therapy, these efficacy data appear rather encouraging. Overall, the characteristics of the patient population included in the 2nd-line cohort of the BYOND and the BODO trial seem relatively similar. However, most pts from BYOND were pre-treated with imatinib (and not with 2nd-generation TKIs). To date, there is only rather limited data available on the efficacy of second-generation TKIs as second-line therapy after failure and/or intolerance of another second-generation TKI.

The run-in dosing strategy evaluated here with bosutinib was successful in less than 60% of our pts, whereas > 40% did not arrive at the 500 mg dose level due to toxicity during the first 3 months of treatment. This led to a median daily dose of bosutinib of 403 mg/day during the first 6 months of treatment which is still higher than in other trials starting with full dose bosutinib. For comparison, median (range) dose intensity in the Ph + CP CML cohort in the BYOND trial amounted to 313.1 (79.7–560.6) mg/day overall; 320.1 (98.4–560.6), 309.4 (79.7–500.0), and 308.0 (125.0–500.0) in the second-, third-, and fourth-line cohorts, respectively [10]. The median dose of the 41 patients who received a minimum of 6 months of bosutinib was 403 mg. Median dose for all 57 patients was 387 mg (range 16–479 mg). Intolerant patients (*n* = 23) had a median dose of 300 mg (range 18–479 mg) while refractory patients (*n* = 34) had a median dose of 391 mg (range 16–478 mg), confirming the findings from other studies. However, the 6 months observation time here was somewhat shorter than in BYOND. In our analysis, we identified weight (expressed as one parameter in BMI) as an independent positive factor associated with successful dosing in (*n* = 34 pts) suggesting that heavier pts were more likely to achieve successful dosing in. A connection between toxicity and BMI has already been described by Brümmendorf et al. [22] in a post hoc analysis of the BFORE trial: nausea (40.9 vs 31.9%), increased alanine (37.6 vs 28.6%), and aspartate aminotransferase (30.2 vs 20.2%) showed differences of about 10% when comparing pts with BMI ≥ 25 vs < 25 with skinnier pts suffering less from these side effects. For hematological toxicities, this effect was reversed with skinnier pts suffering more from thrombocytopenia (30.9 vs 41.2%, BMI ≥ 25 vs < 25). These aspects should be addressed prospectively in future trials.

Overall, and at least in part due to the limited sample size related to the premature study termination, the trial failed to achieve its primary goal, i.e., to prove that a step-in dosing concept indeed significantly reduces the incidence of early-occurring higher grade GI toxicities. Instead and to our surprise, the rate of adverse events overall was comparable to other trials performed in similar scenarios but without the use of step-in dosing. This questions the hypothesis that bosutinib-induced, particularly early-occurring GI toxicities can be mitigated by reduced dosing concepts at least once a starting does of 300 mg is being used. We rather hypothesize that optimized patient management by experienced CML experts may help to optimize bosutinib therapy as ultimately, the AEs only rarely led to permanent treatment discontinuation. Interestingly, median time to first AE of diarrhea in the BODO trial was 23 days, and the median duration of any grade diarrhea was 14 days (range: 1–960). For comparison in study 200, the median time to onset of diarrhea was 2.0 days and grade 3 diarrhea had a median event duration of 3.0 days [3] which might on the one hand raises the hypothesis that via the decreased starting dose we were able to reduce early-onset diarrhea, on the other hand diarrhea intensity and duration seemed to be increased by the run-in dosing concept. The rate of GI toxicity reported here, although based on limited data, seems comparable to former studies (all grade GI toxicity: 90% during the first 6 months; grade 3/4 GI toxicity: 16%) [3, 10]. Importantly however, there were no new or unexpected safety signals or hints towards reduced efficacy or early progression events induced by the run-in dosing schedule observed in our trial.

Dose optimization regimens have already been studied in various bosutinib treatment settings. Kota et al. [12] described similar efficacy results in pts treated with bosutinib either with 400 mg QD or with 500 mg QD in the phase I/II study. Furthermore, bosutinib had initially been tested with 500 mg QD in the first-line setting in the BELA trial [23] but as the primary endpoint was missed, a new trial was initiated investigating a lower dose of bosutinib (400 mg QD) as first-line therapy which was successful [1]. Even in the setting of a starting dose of 400 mg QD further dose reductions to 300 and in some cases even 200 mg QD provided sufficient efficacy while enabling more pts to continue bosutinib treatment with a substantial number of them achieving molecular and cytogenetic responses for the first time after dose reductions [13]. In our study cohort, only 60% of all pts were able to successfully complete the run-in dosing concept, which could lead to the hypothesis that starting with even a lower dose might have been more successful. Indeed, in a non-randomized study by Mita et al. [24], 25 Japanese CML pts were dose-escalated from a starting dose of 100 mg QD with dose increases of 100 mg every 14 days and compared to standard dose 500 mg QD therapy from the beginning. In this trial, the dose escalating regimen enabled all pts to continue bosutinib therapy without AE-related interruptions. In the standard arm, all pts suffered from diarrhea while in the dose-escalating arm, diarrhea was reduced to 73.3% (11 out of 15 pts, all grades). Grade 2 and 3 diarrhea occurred in 2 and 3 pts, respectively. Of note, pooled data from seven different bosutinib trials showed that gastrointestinal (92.8% vs 84.7%) side effects occurred more frequently in Japanese vs. non-Japanese pts [25]. Furthermore, analysis from the BFORE trial revealed higher bosutinib drug levels in Asian vs. non-Asian pts [26] thereby limiting the transferability of tolerability results across ethnic groups. Furthermore, a retrospective analysis of the phase I/II study on bosutinib (study 200) revealed that the incidence of treatment-emergent AEs was lower after dose reduction, particularly for gastrointestinal events [27].

Altogether, the following reasons could be suspected to explain why the dose-increasing regimen investigated here was not able to significantly reduce GI side effects within this study apart from the reduced sample size: 1. The relatively high starting does of bosutinib (i.e., 300 mg QD) which was selected on the basis of available phase 1 data in Caucasians; 2. The relatively short intervals of dose increases (2 weeks) which however was consistent with the approach taken in the Japanese study mentioned above; 3. The study design (one-armed as opposed to a randomized study with a fixed-dose comparator arm) with reduction of toxicity compared to historic controls as primary endpoint; and 4. the relatively high target dose of bosutinib (500 mg QD) reflecting the approved dose in later-line treatment whereas (similar to other TKIs such as nilotinib) the lower dose of (here 400 mg QD) reflects the approved dose in first line.

In summary, this is one of the (if not the) largest cohorts published on the efficacy and safety of a second-generation TKI after intolerance/failure to another 2G-TKI administered in first line. Given the limitations of a single-arm study with premature study closure due to incomplete recruitment, we could not demonstrate an advantage of the step-in dosing concept chosen here to reduce the frequency of grade 2–4 GI toxicity overall. However, using this regimen, bosutinib was able to induce optimal responses according to ELN recommendations [14] in almost two-thirds of pts previously resistant to 2G-TKIs. Furthermore, GI toxicity only very rarely led to treatment discontinuation while liver toxicity remains a considerable challenge. We conclude that our data could not show that bosutinib step-in dosing starting at 300 mg QD and toxicity-related dose adaption leads to significant improvement in early GI toxicity. However, and given that according to the feedback we received, many treating physicians use this strategy in real world; step-in dosing of bosutinib can be considered safe and efficacious as 2nd and 3rd line therapy after failure of previous 2G-TKI therapy.

## Supplementary Information

Below is the link to the electronic supplementary material.

Supplementary file1 (DOCX 33 KB)

## Data Availability

The datasets generated and/or analyzed during the current study are not publicly available but are available from the corresponding author on reasonable request and with permission of the steering committee.

## Abbreviations

*AE*: Adverse event
*AP*: Accelerated phase
*BC*: Blast crisis
*BMI*: Body mass index
*CML*: Chronic myeloid leukemia
*CP*: Chronic phase
*GI*: Gastrointestinal
*HRQoL*: Health-related quality of life
*MMR*: Major molecular remission
*MR*^*4*^* and MR*^*4.5*^: Deep molecular remission
*PROM*: Patient-reported outcome measures
*QD*: Latin: quaque die; once daily
*QoL*: Quality of life
*SAE*: Serious adverse event
*TEAE*: Treatment-emergent adverse event
*TKI*: Tyrosine kinase inhibitor
