# Supplementary material for: Step-in dosing of bosutinib in pts with chronic phase chronic myeloid leukemia (CML) after second-generation tyrosine kinase inhibitor (TKI) therapy: results of the Bosutinib Dose Optimization (BODO) Study
Source: Ann Hematol. 2023 Aug 18;102(10):2741–52. doi: 10.1007/s00277-023-05394-0 (PMC10492675; doi:10.1007/s00277-023-05394-0)
Supplement: Supplementary file 1 — Supplementary file1 (DOCX 33 KB) [file 277_2023_5394_MOESM1_ESM.docx]

**Supplemental file F1: Additional Methods of the BODO trial**

**Endpoints and analyses**

**Additional secondary endpoints**

Key secondary and exploratory endpoints included: i) Efficacy parameters (i.e. MMR, MR^4^, MR^4.5^), ii) patient-reported outcome measures (QoL), iii) the rate of emerging mutations during bosutinib, and iv) overall tolerability. Associations between binary endpoints were examined with the exact Fisher test or the chi-square test. Continuous variables were compared between groups using the Mann-Whitney U test, and for comparisons within groups between different time points, the Wilcoxon signed-rank test was used. Overall survival (OS) and progression free survival (PFS) probabilities were described using the Kaplan-Meier method. Pts not known to have died were censored at the date when they were last known to be alive. PFS time was the time from registration for the study until the date of either disease progression (AP, BC) or death from any cause. Pts without PFS events were censored at the date when they were last known to be alive and without progression. With regards to time-to-event endpoints, groups were compared with the log-rank test or, in case of competing risks, with the Gray test. Apart from confirmatory testing of the primary endpoint, all other analyses were explorative, without adjustment for multiple testing. Estimates were stated together with their 95% CI. The significance level for the two-sided P values was 0.05 for all statistical testing procedures. Analyses were performed using SAS version 9.4 and R version 3.6.3.

**Molecular response**

Molecular response (MR) was assessed in all pts at baseline and every 3 months thereafter upon study inclusion. Levels of BCR::ABL1 transcripts were determined by RQ-PCR testing of peripheral blood and analyzed at one of the reference laboratories (Jena or Mannheim). The percent ratio of BCR::ABL1 transcripts vs. control gene (ABL1) transcripts converted to the international scale were calculated for each sample. For definitions of response criteria in the protocol please see supplementary material.
